# Exploring the Frequency of Anxiety and Depression Symptoms in a Brazilian Sample during the COVID-19 Outbreak

**DOI:** 10.3390/ijerph18094847

**Published:** 2021-05-01

**Authors:** Fabiana Silva Ribeiro, Flávia H. Santos, Luis Anunciação, Lucas Barrozo, Jesus Landeira-Fernandez, Anja K. Leist

**Affiliations:** 1 Department of Social Sciences, University of Luxembourg, L-4366 Esch-Sur-Alzette, Luxembourg; anja.leist@uni.lu; 2 School of Psychology, University College Dublin, D04 V1W8 Dublin, Ireland; flavia.santos@ucd.ie; 3 Department of Psychology, Pontifical Catholic University, 22541-041 Rio de Janeiro, Brazil; luisfca@gmail.com (L.A.); landeira@puc-rio.br (J.L.-F.); 4 Institute of Psychology, Federal University of Rio de Janeiro, 21941-901 Rio de Janeiro, Brazil; lucas.psic.rj@gmail.com

**Keywords:** depression, anxiety, social distancing, Brazil, COVID-19

## Abstract

The COVID-19 pandemic is a public health emergency of international concern, and the main measures to contain the spread of the coronavirus causing COVID-19 were social distancing, quarantine, and self-isolation. Although these policies are effective in containing the spread of the virus, they might represent a challenge to psychological well-being, increasing levels of depressive and anxiety-related symptoms. Aims: We explored the frequency of anxiety and depression symptoms during COVID-19 restrictions and associations with sociodemographic factors in a Brazilian sample. Method: Data of a total of 936 Brazilian adults (68.2% women) aged 18 to 77 years old (M = 38.95, SD = 13.91) were collected through an online survey. Results: In general, we observed a frequency of 17.36% for severe anxiety and 66.13% for severe depression symptoms, in which younger participants (18–39 years old) and women showed higher scores in anxiety and depression scales compared to older age groups. Logistic regressions showed that women were more likely to present severe symptoms of anxiety (20.4%) compared to men (10.9%), as well as respondents in the educational sector (24.3%) compared to those in the health sector (10%). Conclusions: We highlight the importance of mental health professionals in developing strategies to help younger adults to mitigate the effects of social restriction.

## 1. Introduction

The COVID-19 pandemic is a public health emergency of national and international interest. Confinement measures represent the best way to prevent the spread of the virus, and yet, a challenge to psychological well-being [1]. During the COVID-19 pandemic, the fear of getting sick [2], the possible loss of a loved one [3], income reductions [4], as well as the experience of social isolation, intensified vigilance for potential threats, which, consequently, may influence the emergence of anxiety symptoms [5,6]. Besides, anxiety can trigger depressive symptoms by activating persistent worries, negative expectations, and thoughts about death [7]. In the case of predisposition, prolonged exposure to adversity, and lack of treatment, anxiety symptoms can become chronic, leading to depression or other mental disorders [8]. According to Beck [9], depressive symptoms are accompanied by the absence of positive emotions and the pervasive presence of feelings of sadness, negative thoughts of the self, and the future. 

In fact, the results of studies assessing the immediate psychological impacts and associated factors during the COVID-19 outbreak among China’s general population showed that 28.8% of respondents had rates corresponding to moderate to severe anxiety symptoms, and 6.5% reported moderate to severe depressive symptoms. Greater psychological effects of the pandemic were associated with being a woman or/and a student [1]. Another study carried out in China in December 2019 in a university student sample reported that 0.9% of respondents were showing symptoms of severe anxiety, while 21.3% showed mild anxiety [10]. 

Similarly, studies performed in Europe revealed an increased prevalence of anxiety and depression in younger populations, specifically in Spain [11], Italy [12,13], and Ireland [14]. These impacts on mental health could have been caused by social isolation and loneliness, which might affect respondents more susceptible to mental illness, since symptoms seem to worsen in the absence of interpersonal communication [15,16]. 

Although more developed countries have relief funds to support their citizens during the pandemic, another essential finding was, for instance, in China, that a protective factor was the stability of family income, since those participants without a fixed family income showed increased anxiety [10], which might be related to increased economic obstacles [17]. Similarly, in a study carried out in the U.S. with young adults, those participants with household savings below $5000 showed an increased risk for developing anxiety symptoms [18]. Furthermore, in an Irish representative sample, loss of income due to COVID-19 proved to be a risk factor for depression and anxiety [14]. This is particularly important, since high-intensity stressors, such as low socioeconomic status and symptoms of depression, can be a potential trigger for suicidal ideas and suicide attempts [19], in addition, depression symptoms seem to affect a larger proportion of young women than men [20]. In fact, inequalities in income, education and wealth have proved to be an essential determinant of depression [21] and anxiety [22].

According to the last World Health Organization [23] report, the Brazilian population, compared to that of the rest of the world, presented one of the highest prevalence of anxiety (9.3%) and depression disorders (5.8%), especially compared to developed countries. For this reason, in the context of the COVID-19 pandemic, the prevalence of anxiety and depression symptoms is expected to be higher than in high-income countries due to increased inequalities, a vulnerable population, and limited resources to deal with the pandemic [24].

Beyond the inherent socioeconomic inequalities aggravated by the measures of confinement, since the beginning of the COVID-19 outbreak, Brazil’s public health messages on the severity of the virus were implemented slowly and with lower strictness than in other countries [25], and “fake news” in social media was widely disseminated. The lack of reliable and up-to-date information about recovery cases and possible treatments might also increase anxiety [1]. In fact, uncertainty levels may be compounded by inaccurate or misleading information about the outbreak on social media [26].

Indeed, a study carried out in Brazil by Campos et al. [27] with 12,196 people showed that 61.3% of respondents were depressed, and 44.2% suffered from anxiety symptoms. Being young (<33 years), a woman, and having a lower economic status were associated with higher depressive and anxiety symptoms. The authors argue that, in addition to the pandemic’s unpredictability, Brazil’s economic and political situations contribute to enhanced vulnerability, especially for those who lack the skills to manage emotional issues developed in a pandemic context.

Another study performed by Serafim et al. [28], also in Brazil and with 3000 people, showed that 46.4% reported symptoms of depression, while 39.7% reported symptoms of anxiety. These symptoms were higher in women, students, patients with chronic diseases, and people who had contact with others diagnosed with COVID-19, possibly because respondents might adopt maladaptive strategies to cope with the pandemic, such as thought rumination, social withdrawal, and/or substance abuse. Findings of Goulart et al. [29] corroborate higher evidence for symptoms of severe anxiety in 81.9% and severe depression in 68%, which are both associated with young groups, being a woman, having a low income, a lower formal education, and a more extended confinement period. Consistently, Passos et al. [30] identified in Portuguese and Brazilian participants that being in social isolation was significantly related to increased levels of depression.

Finally, Feder et al. [31], through a retrospective cohort study, including a Brazilian sample, aimed to evaluate the prevalence of depression and anxiety symptoms before and during the COVID-19 pandemic, and to explore possible risk factors associated with these symptoms. They observed a sharp increase in anxiety, from 3.9% to 29.1%, and depression symptoms, from 4.5% to 37.8%. As in the above-quoted studies, higher depression and anxiety symptoms were observed in women, younger participants, participants diagnosed with chronic disease, and those economically affected by restriction measures. The authors claim that fear of unemployment and future insecurity among younger adults could potentially be responsible for worse mental health scores

### Our Study

Although we observed published studies, including Brazilian samples exploring the frequency of anxiety and depression, studies examining whether participants were tested for COVID-19 and those looking at economic changes during social restriction measures and their impact on mental health are scarce.

For this reason, we sought to evaluate the frequency of anxiety and depression symptoms in a Brazilian sample; to assess mental health states in different age groups and whether participants with changes in their income would present differences in depression and anxiety symptoms to those whose income did not change due to social restriction measures. Furthermore, we aimed to observe how many participants were tested for COVID-19. Finally, we investigated possible factors influencing severe anxiety and depression symptoms during the COVID-19 containment measures in the spring of 2020. 

We hypothesized that the frequency of anxiety and depression symptoms would be high due to misinformation regarding the pandemic [32]. Moreover, we expected younger respondents, women, and participants from low socioeconomic backgrounds, represented by lower income and those who had their income affected by the social distancing, to report higher odds of anxiety and depression compared with respondents from higher socioeconomic backgrounds. We further hypothesized that respondents who are less able to comply with social distancing (i.e., having to leave the house more often), or having less frequent in-person social contact would show higher odds of anxiety and depression. Finally, professionals from the health sector would present higher levels of anxiety and depression due to direct contact with the disease.

## 2. Materials and Methods

### 2.1. Ethical Statement

This study used data from two surveys carried out in Brazil that have received ethical approval by the responsible Ethics Committees, which were São Paulo State University “Júlio de Mesquita Filho” Bauru Campus (Process: 4.021.098) and Gaffrée and Guinle University Hospital, Rio de Janeiro (Process: 4.125.060). We obtained electronic informed consent from all participants before they began to fill in the online survey. 

### 2.2. Sample Size 

We estimated the sample size using Raosoft software, using a conservative assumption, we applied a level of confidence of 95%, with a 50% response distribution and an error margin of 5%. The minimum sample size recommended was 385 participants.

### 2.3. Participants

Participants of both studies answered the questionnaires and scales anonymously through an online survey running from 27 April to 5 July 2020, and they received no compensation for it. We applied a snowball sampling approach since it was not possible to establish the sample’s randomness due to the need of starting data collection as soon as possible. In total, 981 respondents started the survey; however, 8 of them were under 18 years old and were excluded from the final analysis. Only participants who reported living in a city with social distance restrictions in place were included in this study, which led to the exclusion of *n* = 24 respondents. Thus, a total of 936 participants who completed at least 90% of the survey were included in the analysis. All respondents lived in Brazil, with 86.15% in the southeast states, 5.87% in the northeast, 4.66% in the south, 1.88% in the north, and 1.44% in the center west.

### 2.4. Material

We used two different surveys, one released in São Paulo and the other in the Rio de Janeiro state. However, the questionnaire items analyzed in this study were collected with similar wording, and the depression and anxiety scales were the same. Aiming to unify the datasets with varied formats, we harmonized the studies of both data, transforming it into one cohesive dataset with the variables of interest for this study. The online survey was available through the Google Forms platform and comprised the three sections described below.

#### 2.4.1. Demographic Questionnaire

This questionnaire contained questions on age, marital status, gender, living arrangements, nationality, state, formal education, professional sector, and socioeconomic factors such as monthly income.

#### 2.4.2. Questionnaire Related to Epidemiological Factors and Behaviors Due to COVID-19

This questionnaire contained questions adapted from the World Health Organisation guidelines [33]; for instance, number of times leaving the house during social distancing and frequency of in-person and online social contacts. Additionally, we asked whether respondents had experienced any changes in salary after the beginning of the confinement measures due to COVID-19, and finally, whether they had been tested for COVID-19.

#### 2.4.3. Mental Health Scales

Two mental health scales were included to assess anxiety and depression. The Generalized Anxiety Disorder (GAD-7) [34] assesses symptoms of anxiety with 7 items, the responses to which are, on a 4-point Likert scale, “not at all,” “several days,” “more than half the days”, or “nearly every day”. Participants were classified with severe symptoms of anxiety when their summary score was ≥ 15 points. A validated Brazilian–Portuguese version was used [35]. The GAD-7 showed good internal consistency and reliability (Cronbach’s alpha of 0.91) in this study. The Center for Epidemiologic Studies Depression Scale (CES-D) [36] comprises 20 items that assess depressive symptoms, and it is able to be used across various age groups in epidemiological studies. The scores of items 4, 8, 12, and 16, with positive wording, are reversed. The final score ranges from 0 to 60 points and corresponds to the sum of the scores of all answers to the items. Participants were classified with severe symptoms of depression when they presented a summary score of >15 points. A validated Brazilian–Portuguese instrument was used [37,38]. The CES-D had a Cronbach’s alpha of 0.90 in our study, showing good internal consistency and reliability.

### 2.5. Procedure

Participants were recruited through social media (Twitter, Facebook, and Instagram). Those who clicked on the link provided in the posts were redirected to the questionnaire hosted on Google Forms. Firstly, respondents read the informed consent and agreed to participate by clicking “I agree to participate”. An e-mail address created for this study was available to participants if they had any questions or concerns, but no events were reported. Then, participants were asked to fill in the three sections of the survey consecutively. Participants took about 20–30 min to finalize the survey.

### 2.6. Statistical Analysis

First, descriptive analyses were conducted to describe the demographic characteristics of the sample and the frequency of social distancing behavior (i.e., number of times leaving the house or number of times leaving home), and changes in socioeconomic factors (i.e., income) using Chi-square tests (χ^2^). Second, we confirmed the normality of continuous variables and carried out *t*-tests and one-way ANOVAs with Bonferroni for multiple comparison post-hoc tests to analyze the differences between sociodemographic characteristics in anxiety and depressive symptoms. Third, we performed χ^2^ tests to compare possible differences among age groups and sociodemographic characteristics. Finally, we performed two separated multiple logistic regression models to investigate whether sociodemographic or COVID-19 related variables would influence severe anxiety and depression classifications during the COVID-19 pandemic. To select the variables that would be included in these models, simple logistic regressions were carried out with each sociodemographic and COVID-19-related variable. To provide a full account of potential covariates of anxiety and depression, all variables with significance levels equal to *p* < 0.05 were included in the multiple logistic regression models. We reported the odds ratio (OR), 95% confidence interval (95% CI), and *p* values. All statistical analyses above cited were conducted through STATA (release 16, StataCorp LP, College Station, TX, USA:) software.

## 3. Results

The demographic characteristics of our sample are displayed in Table 1. From the total cohort of 936 participants, 68.16% were women, and respondents were 18 to 77 years old (M = 38.95, SD = 13.91). Most participants had post-secondary education (46.79%) or post-graduation (40.06%). Moreover, 21.47% worked in the health sector. In general, we observed a frequency of severe depression symptoms of 66.13% and a frequency of 17.36% for severe anxiety symptoms.

### 3.1. Testing and Sociodemographic Factors during COVID-19 Pandemic

A total of 4.54% (*n* = 42) of the sample reported that they had been tested for COVID-19. Regarding the pandemic’s socioeconomic impact, 15.93% (*n* = 147), respondents were not receiving their salaries due to COVID-19 pandemic restrictions. Those respondents employed in the education sector reported leaving the house fewer times per day (37.7%) compared to other professional sectors (health: 16.42%; others: 12.64%), χ^2^ = 24.97, *p* < 0.001.

We also observed that respondents with post-secondary education (20.60%) were more likely to present changes in their salaries due to COVID-19 measures compared to post-graduates (10.51%), χ^2^ = 15.60, *p* < 0.001.

### 3.2. Comparisons of Anxiety and Depression Scores by Sociodemographic and COVID-19 Characteristics

The analyses performed with *t*-tests, with sex as the independent variable and depression and anxiety symptoms as dependent variables, revealed that women reported higher scores for both scales (M = 22.68; SD = 12.35 for depression; M = 9.19; SD = 10.81 for anxiety) compared to men (M = 19.46; SD = 10.81 for depression; M = 7.35; SD = 5.14 for anxiety), *t* (930) = 4.04, *p* < 0.001, *t* (930) = 4.90, *p* < 0.001, respectively. Regarding the *t*-tests for changes in income due to COVID-19 and depression and anxiety symptoms, we observed no differences, *t* (931) = −1.79, *p* = 0.07*, t* (931) = −0.32, *p* = 0.75, respectively.

In the following, we report group differences and bivariate associations with *p*s < 0.005. The ANOVAs results are displayed in Table 2, and pairwise comparisons revealed that the youngest groups (18–29 years) scored higher for anxiety and depression symptoms compared to other age groups (*p*s < 0.001). Individuals from the professional education sector revealed higher depression symptoms in contrast with unemployed, health and other sectors (*ps* < 0.001). Respondents who reported they were “working at the moment” showed lower depression scores than those not working (*p* = 0.002). 

Moreover, χ^2^ tests showed that younger participants (67.63%) were more likely to have post-secondary education than the other age groups (χ^2^ (8) = 115.78, *p* < 0.001). Younger people were more likely to be single, at 85.56%, χ^2^ (8) = 115.78, *p* < 0.001. However, the young age group was more likely to live with their parents (63.54%) when compared to the other age groups (36.46%), χ^2^ (12) = 257.33, *p* < 0.001. Furthermore, younger groups were more likely to receive 1–3 wages compared to the other age groups, χ^2^ (12) = 86.61, *p* < 0.001, as well as to be unemployed (14.39%) compared to other age groups, χ^2^ (12) = 74.14, *p* < 0.001. Finally, young people were less likely to be working in comparison to those who were 30–59 years old, χ^2^ (4) = 60.80, *p* < 0.001.

Regarding sex, 41.22% of women reported not leaving home compared to 27.37% of men, χ^2^ (3) = 18.01, *p* < 0.001. In addition, more women reported working in a health profession (25.39%) compared to men (13.13%), χ^2^ (3) = 30.28, *p* < 0.001.

### 3.3. Association of Depression and Anxiety Symptoms with Covariates 

In Table 3, we report odds ratios (OR) and 95% confidence intervals for the explanatory variables resulting from the logistic regression models. Aiming to understand whether respondents from lower socioeconomic backgrounds would report higher rates of severe anxiety and depression symptoms, we ran separate models, with anxiety and depression symptoms as outcomes. We adjusted for sociodemographic characteristics.

As shown in Table 3, we observed that women were more likely to present severe symptoms of depression, as well as anxiety, compared to men. Moreover, we observed that older respondents (>50 years old) were less likely to present severe levels of anxiety and depression symptoms than younger respondents (18–29 years old). Respondents with higher income (>10 wages) were less likely to present severe symptoms of depression compared to those receiving 1–3 wages. Finally, those participants leaving home more than twice during social distancing measures were less likely to present severe depression symptoms compared to those who reported not leaving home.

## 4. Discussion

The main goal of this study was to evaluate the frequency of anxiety and depression symptoms of a Brazilian sample during COVID-19 confinement measures and explore whether socioeconomic factors would be associated with symptoms of anxiety and depression. As predicted, our findings indicated a high frequency of anxiety symptoms and a very high frequency of depression affecting most of the Brazilian sample during the COVID-19 pandemic. These results are consistent with studies assessing mental health in the Brazilian population [27,28,29,31]. Congruent with a retrospective cohort study in Brazil [31], younger people and women exhibit a higher frequency of anxiety and depression, which could be explained by a higher vulnerability in dealing with social distancing measures [31]. Delivering data regarding mental health in Brazil, in which the government has provided limited information about the pandemic and the number of deaths has been among the highest in the world [39], might help develop policies and plans actions concerning mental health during and in upcoming outbreaks.

An alarming finding of this study was that only a small minority of the sample (4.54%) was tested for COVID-19. This result unveils underestimated numbers of cases and corroborates that Brazilian testing rates are among the lowest in the world, with approximately 3462 people tested per 1 million inhabitants during the time of data collection [25]. This underreported data leads to difficulties in the implementation of effective healthcare responses.

Although older people are the primary mortal victims of the COVID-19 pandemic, our results suggested that the mental health of young people is highly impacted by a scenario that combines low income, lack of autonomy and reduced social interaction [27,28,29,31,40]. Congruently, in this study, we observed higher scores of anxiety and depression symptoms for the younger age group, as well as positive associations through logistic regression. These findings can be interpreted as a result of uncertainty associated with professional and personal futures [27]. Furthermore, one should have in mind that those 18–29 years old are in a phase in which they are developing autonomy, leaving their parents’ homes, and building interpersonal relationships; all these factors were paused for an uncertain period of time [40]. In fact, we evidenced that younger people were those with post-secondary education, single, living with parents, receiving 1–3 monthly wages, and more likely to be unemployed. All factors proved to influence mental health quality [27,29], in addition to promoting feelings of loneliness and undeveloped strategies to cope with social isolation [27].

Moreover, women presented higher prevalence of anxiety and depression symptoms, which is also congruent with studies performed in Brazil [27,28,29,30,31] and other countries [1,11,13,14]. We observed that women were more likely to not leave home compared to men, which was already associated with fear, boredom, frustration, and anger [41]; besides, women seem to be more likely to ruminate on negative thoughts than men, which can prevent the development of healthy, adaptive coping strategies [42].

The prevalence of severe depressive symptoms was higher compared to studies running during the COVID-19 pandemic in high-income countries [6,7,8,9,11,12,13,14], which could suggest that participants had difficulties in dealing with the emotional factors related to the COVID-19 outbreak in, for instance, exposure to uncontrollable experiences [43] that can cause helplessness and lack of motivation, leading to increased symptoms of depression [44]

Our study detected a prevalence of 17.36 % of severe anxiety symptoms, which, compared to two studies carried out in China, was lower than the anxiety rate of 35% [26,45] but comparable to lower anxiety rates from 12.9% to 16.3% [1,46,47]. These discrepancies could be explained by the different instruments or by context factors, or both. Although the data collection did not allow us to ask about subjective experiences during the pandemic, the sociodemographic differences taken into account in our study suggest that, in particular, those economically affected by pandemic control measures were at risk of severe anxiety- and depression-related symptoms. Another, unobserved reasons for the higher reported symptoms of depression may be the fear of falling ill or dying from the virus, as well as losing loved ones or perceived insecurity about prospects [48,49]. The lack of strict containment measures in Brazil [50], unlike what has been observed in China, may have additionally increased worries and feelings of insecurity.

As governmental directives were ambiguous, we observed that only 36.79% of the respondents were following social distancing measures, which explained the exponential spread of the virus, regardless of the new variant. In some countries, government attitudes and campaigns led to a sense of a collective identity in which individual actions account for the protection of the entire society [51]. That is, the more conscious a society is of the consequences of the virus’s spread, the more compliant with protective measures citizens become. Furthermore, we found that, for respondents in more strict social isolation practices (i.e., participants who reported not leaving their houses during the day), severe depression symptoms were higher than for those who reported less strict social isolation practices. This result is in line with a Brazilian study revealing higher feelings of loneliness for those following confinement restrictions, increasing psychological distress [52]. It cannot be ruled out that compliant citizens, on top of the impact of loneliness and isolation, were overly worried about the majority who did not respect protective measures and the irredeemable consequences of their actions.

By contrast, educational professionals presented higher levels of anxiety and depressive symptoms compared to professionals in the health sector. This result may be due to the uncertainty and stress among teachers because of the abrupt closure of schools and the need to adapt to new working conditions, such as distance education [53]. Moreover, the professionals in the health sector who responded to our survey were not workers on the frontline of COVID-19, since the majority of our respondents were psychologists, probably working from home.

### 4.1. Strengths and Limitations

The strength of our study was a timely data collection and a large sample of respondents from the most populous regions of Brazil [54], which is noteworthy since the research in this country is underfunded and presents serious turmoil since the beginning of the COVID-19 pandemic [55]. Moreover, we also investigated the changes caused by social distancing measures on monthly wages due to social distancing restrictions.

It would have been ideal to have in-person psychiatric assessments for the clinical diagnosis of anxiety and depression. However, pandemic restrictions did not allow for such a procedure at the time. To partly compensate for this shortcoming, we only chose validated scales for the assessment of anxiety and depression.

One of the limitations of the study is a lack of pre-pandemic levels of depression and anxiety for the sample. Furthermore, the randomization of our sample was not possible due to the use of a convenience online survey to prevent infections, which could cause sample bias, and which resulted in a sample that was not balanced in terms of residence across states of Brazil. In fact, we have to recognize that our sample was highly educated and in better socioeconomic standing than a population-representative sample. Even though some participants had their economic situation affected, they were in the minority, and likely underrepresented in our sample. Despite earlier evidence that showed higher anxiety prevalence, particularly for those with lower socioeconomic standing [15,16], we found elevated levels of anxiety in people with a good socioeconomic standing. Finally, as our study was conducted within a four-month period and the COVID-19 pandemic is marked by a volatile scenario, it is possible that different periods of social distancing could have impacted the mental health results of our study.

### 4.2. Implications for Policy Making

Finally, the results of this study reinforce the importance of paying special attention to young adults, since, for those affected by mental health disorders, it may go along with other negative unintended and long-term harmful consequences, such as a decreased ability to work and increased healthcare costs. We suggest that younger adults could benefit from mental health care through psychoeducation programs such as resilience training. For example, coping strategies can promote behaviors that protect against the negative symptoms caused by the necessity to follow social distancing rules [56,57].

## 5. Conclusions

In conclusion, the findings underline the importance of mental health clinicians and researchers in developing strategies to mitigate the psychological impact of restrictive pandemic control measures, mainly on young adults. Providing additional psychological support measures should be a public health priority during and in the aftermath of the COVID-19 pandemic.

## Figures and Tables

**Table 1:** Demographic characteristics of the sample.

Variable	*n* (%)
Age groups	(*n* = 936)
18–29 years	278 (29.70)
30–39 years	267 (28.53)
40–49 years	152 (16.24)
50–59 years	142 (15.17)
>60 years	97 (10.36)
Age—*M(SD)*	38.95 (13.91)
Education	(*n* = 936)
Secondary education	123 (13.14)
Post-secondary education	438 (46.79)
Post-graduation	375 (40.06)
Professional sectors	(*n* = 936)
Unemployed	60 (6.41)
Education	228 (24.36)
Health	201 (21.47)
Other sectors	447 (47.76)
Monthly Income: number of minimum wages	(*n* = 925)
1–3	295 (31.89)
3–6	240 (25.95)
6–10	184 (19.89)
>10	206 (22.27)
Marital status	(*n* = 935)
Single	422 (45.13)
Married/ de facto union	435 (46.52)
Divorced	68 (7.27)
Widow	10 (1.07)
Living arrangement	(*n* = 931)
Alone	103 (11.06)
Parents	277 (29.75)
Family	532 (57.14)
Friends and colleagues	19 (2.04)
Working at the moment	
No	257 (27.55)
Yes	676 (72.45)
Wage changes due to the COVID-19	
No	776 (84.07)
Yes	147 (15.93)
Number of times leaving the house per day during social distancing measures	
None	344 (36.79)
1 time	353 (37.75)
2 times	135 (14.44)
>3 times	103 (11.02)
Number of in-person contacts	
None	244 (26.07)
1–2	436 (46.58)
>3	256 (27.35)
Number of online social contacts	
None	111 (11.88)
1–2	213 (22.81)
>3	610 (65.31)
Anxiety classification	
No anxiety	771 (82.64)
Severe anxiety	162 (17.36)
The Generalized Anxiety Disorder—*M(SD)*	8.62 (6.64)
Depression classification	
No depression	316 (33.87)
Severe depression	617 (66.13)
The Center for Epidemiologic Studies Depression Scale—*M(SD)*	21.68 (11.98)

Note: One minimum wage ∼ USD 195.69; M = mean; SD = Standard Deviation.

**Table 2:** Comparisons of anxiety and depression scores according to sociodemographic and COVID-19 characteristics.

	Anxiety Scores *M (SD)*	*F_(DF)_*	*p*	Depression Scores *M (SD)*	*F_(DF)_*	*p*
Age groups		***F_(4928)_***			***F_(4928)_***	
18–29 years	10.12 (5.64)	16.43	<0.001	26.10 (12.52)	22.15	<0.001
30–39 years	9.13 (5.35)			22.25 (10.64)		
40–49 years	8.47 (6.69)			19.99 (11.02)		
50–59 years	6.84 (5.50)			18.16 (11.87)		
>60 years	5.72 (4.76)			15.29 (10.61)		
Education		***F_(2930)_***			***F_(2930)_***	
Secondary	9.75 (5.77)	6.97	<0.001	25.22 (12.58)	16.02	<0.001
Post-secondary	8.96 (5.73)			22.84 (11.99)		
Post-graduation	7.84 (5.39)			19.18 (11.29)		
Professional sectors		***F_(3929)_***			***F_(3929)_***	
Unemployed	9.18 (5.19)	5.99	<0.001	24.67 (13.18)	7.13	<0.001
Education	9.58 (5.85)			23.29 (12.41)		
Health	7.33 (5.02)			18.62 (10.53)		
Other sectors	8.63 (5.75)			21.85 (11.96)		
Monthly Income: number of minimum wages		***F_(3918)_***			***F_(3918)_***	
1–3	9.94 (5.89)	9.32	<0.001	25.30 (12.39)	20.54	<0.001
3–6	8.50 (5.76)			22.23 (12.13)		
6–10	8.10 (5.04)			20.67 (11.44)		
>10	7.41 (5.26)			17.16 (9.90)		
Marital Status		***F_(3928)_***			***F_(3928)_***	
Single	9.43 (5.62)	5.96	<0.001	24.36 (11.75)	13.41	<0.001
Married/ de facto union	8.06 (5.66)			19.30 (11.75)		
Divorced	7.76 (5.08)			20.87 (11.34)		
Widow	5.50 (4.25)			20.40 (13.02)		
Living arrangement		***F_(3, 924)_***			***F_(3, 924)_***	
Alone	7.32 (5.23)	5.26	<0.001	20.20 (10.47)	12.48	<0.001
Parents	9.63 (5.63)			25.37 (12.27)		
Family	8.35 (5.67)			20.19 (11.82)		
Friends/colleagues	8.79 (5.40)			20.53 (9.23)		
Working at the moment	***F_(1928)_***			***F_(1928)_***		
No	9.15 (5.57)	3.08	0.08	23.71 (12.81)	9.90	0.002
Yes	8.42 (5.65)			20.95 (11.58)		
Number of times leaving the house per day during social distancing measures		***F_(3928)_***			***F_(3928)_***	
None	9.03 (5.69)	3.13	0.02	23.20 (12.39)	5.66	<0.001
1 time	8.85 (5.60)			21.94 (11.97)		
2 times	7.49 (5.53)			19.30 (10.39)		
>3 times	7.92 (5.57)			18.83 (11.80)		
Number of in-person contacts	***F_(2930)_***			***F_(2930)_***		
None	7.98 (5.70)	2.17	0.11	20.30 (11.08)	3.45	0.03
1–2	8.90 (5.58)			22.72 (12.31)		
>3	8.73 (5.63)			21.24 (12.11)		
Number of online social contacts	***F_(2928)_***			***F_(2928)_***		
None	9.17 (5.76)	1.31	0.27	23.01 (13.32)	1.08	0.34
1–2	8.96 (5.41)			22.15 (11.36)		
>3	8.42 (5.68)			21.34 (11.90)		

**Table 3:** Results of multivariate logistic regression analyses, adjusted for socioeconomic variables and social distancing, on severe anxiety and depression symptoms (*n* = 857).

Depression	Odds Ratio (CI)	*p*	Anxiety	Odds Ratio (CI)	*p*
Gender					
Women (reference)					
Men	0.66 (0.57–1.48)	0.01		0.50 (0.32–0.79)	0.003
Profession sector					
Education (reference)					
Health	0.66 (0.43–1.04)	0.07		0.44 (0.24–0.79)	0.007
Others	1.05 (0.71–1.56)	0.81		0.77 (0.50–1.19)	0.24
Age groups					
18–29 years (reference)					
30–39 years	0.92 (0.57–1.48)	0.73		0.61 (0.36–1.02)	0.06
40–49 years	0.66 (0.39–1.14)	0.14		0.68 (0.37–1.25)	0.21
50–59 years	0.38 (0.22–0.65)	<0.001		0.44 (0.23–0.86)	0.02
>60 years	0.19 (0.10–0.36)	<0.001		0.17 (0.06–0.51)	0.002
Education					
Primary-secondary (reference)					
Post-secondary	0.91 (0.50–1.62)	0.74		0.84 (0.47 -1.50)	0.55
Post-graduation	0.71 (0.39–1.32)	0.29		0.78 (0.40–1.53)	0.47
Income: number of minimum wages					
1–3 (reference)					
3–6	0.66 (0.42–1.02)	0.06		0.76 (0.47–1.25)	0.28
6–10	0.77 (0.48–1.25)	0.29		0.69 (0.39–1.21)	0.20
>10	0.53 (0.34–0.87)	0.01		0.63 (0.34–1.15)	0.13
Number of times leaving the house during social distancing measures					
None (reference)					
1 time	.78 (0.54–1.13)	0.19		0.47 (0.17–1.29)	0.14
2 times	0.54 (0.34–0.86)	0.009		0.92 (0.34–2.54)	0.87
>3 times	0.54 (0.32–0.90)	0.02		1.62 (0.57–4.58)	0.36
Living arrangements					
Alone (reference)					
Parents	1.56 (0.88–2.79)	0.13		1.60 (0.71–3.58)	0.26
Family	0.89 (0.54–1.46)	0.65		1.83 (0.85–3.94)	0.12
Friends and colleagues	1.15 (0.36–3.72)	0.81		2.17 (0.54–8.71)	0.28

Note: One minimum wage ∼ USD 195.69.

## Data Availability

The data presented in this study are available on request from the corresponding author. The data are not publicly available due to privacy.
